# The Relevance of Interoception in Chronic Tinnitus: Analyzing Interoceptive Sensibility and Accuracy

**DOI:** 10.1155/2015/487372

**Published:** 2015-10-25

**Authors:** Pia Lau, Miriam Miesen, Robert Wunderlich, Alwina Stein, Alva Engell, Andreas Wollbrink, Alexander L. Gerlach, Markus Junghöfer, Thomas Ehring, Christo Pantev

**Affiliations:** ^1^Institute for Biomagnetism and Biosignalanalysis, University Hospital of Münster, Malmedyweg 15, 48149 Münster, Germany; ^2^Institute of Psychology, University of Münster, Fliednerstraße 21, 48149 Münster, Germany; ^3^Institute for Physiological Psychology, University of Bielefeld, Universitätsstraße 25, 33615 Bielefeld, Germany; ^4^Institute of Clinical Psychology and Psychotherapy, University of Cologne, Pohligstraße 1, 50969 Cologne, Germany; ^5^Department of Psychology, LMU Munich, Leopoldstraße 13, 80802 Munich, Germany

## Abstract

In order to better understand tinnitus and distress associated with tinnitus, psychological variables such as emotional and cognitive processing are a central element in theoretical models of this debilitating condition. Interoception, that is, the perception of internal processes, may be such a psychological factor relevant to tinnitus. Against this background, 20 participants suffering from chronic tinnitus and 20 matched healthy controls were tested with questionnaires, assessing interoceptive sensibility, and participated in two tasks, assessing interoceptive accuracy: the Schandry task, a heartbeat estimation assignment, and a skin conductance fluctuations perception task assessing the participants' ability to perceive phasic increases in sympathetic activation were used. To test stress reactivity, a construct tightly connected to tinnitus onset, we also included a stress induction. No differences between the groups were found for interoceptive accuracy and sensibility. However, the tinnitus group tended to overestimate the occurrence of phasic activation. Loudness of the tinnitus was associated with reduced interoceptive performance under stress. Our results indicate that interoceptive sensibility and accuracy do not play a significant role in tinnitus. However, tinnitus might be associated with a tendency to overestimate physical changes.

## 1. Introduction

Tinnitus affects up to 40% of the population in Western countries at least temporarily [1]. One to three percent of the general population report a significant reduction in their quality of life due to their tinnitus, for example, through its effect on sleep and/or mood [2]. It is widely assumed that tinnitus is a result of maladaptive cortical plasticity [3]. Yet psychological constructs are believed to mediate this process and are especially tied to the distress perceived because of the tinnitus [4–6]. Current psychological models of tinnitus assume a neuronal basis of the tinnitus and in addition focus on the interplay of different psychological processes explaining the perceived distress [7]. For example, McKenna et al. [7] propose that tinnitus distress starts with the detection of tinnitus. Then, a vicious cycle of negative automatic thoughts, detrimental safety behaviors, selective attention, and monitoring is triggered. This model draws distinctively from models of other mental disorders such as panic disorder. In the case of panic attacks, small internal changes, for example, of the heartbeat, trigger a similar dysfunctional circuit and in the end result in panic attacks (cf. [8]) Hence, one risk factor for panic disorder is interoception [9]. Interoception is defined as sensitivity to internal stimuli which originate from the body itself [10]. Interoception is also connected to other mental disorders, including general anxiety disorder, bulimia nervosa, anorexia nervosa, and somatoform disorders [11–14]. In addition, interoception has been shown to be linked to psychological variables, such as emotional experience, emotional memory processes, and alexithymia [14–16], which are also discussed in the context of tinnitus (e.g., [17, 18]).

Tinnitus* per se* is a process of interoception as it is attention toward internal percept. Whether interoception can be assumed to be a dysfunctional factor for chronic tinnitus however is still obscure. Next to the overlap of etiological models, psychotherapeutic aspects for mental disorders and tinnitus have common characteristics: an important intervention in evidence-based treatment of panic disorder is interoceptive exposure, which includes purposely evoking internal stimuli (e.g., hyperventilating and running steps to increase the heartbeat) in order to make the patients learn that those internal signals are not harmful [19]. Similarly, intentionally focusing on the tinnitus is a strategy used in current treatments for tinnitus [20]. This intervention showed a significant reduction in tinnitus related distress [21] which points towards a meaningful connection between interoception and chronic tinnitus.

More evidence for a connection between chronic tinnitus and interoception comes from the field of neuroscience: the right anterior insula is activated in interoceptive processes likewise in tinnitus sufferers, especially if they are highly distressed [22–26]. Taken together, the current research concerning tinnitus offers hints for a connection between chronic tinnitus and interoception, but this question has never been addressed directly. Hence, this study can be seen as a first step towards a better understatement of the putative role of interoception in tinnitus.

Current research suggests that interoception exhibits a threefold structure: interoceptive sensibility, accuracy, and awareness [27]. Interoceptive sensibility is regarded as the subjective perception of interoception measured through questionnaires or interviews. Interoceptive accuracy, sometimes also named sensitivity, is the objective measurement of the accurate detection of internal processes. Finally, interoceptive awareness is described as higher-order component in interoception and covers more a metacognitive understanding of interoception, for example, the knowledge about the accuracy of the own interoceptive perception. As the latter is difficult to measure (cf. [28]) and our study focused on the basic aspects of interoception, we collected data on the first two components, namely, interoceptive sensibility and accuracy.

A standard procedure to operationalize interoceptive accuracy is using the Schandry task [29]. Participants have to report on all heartbeats felt during a signaled period of time. The participants have to rely solely on their feeling while no auxiliary means are allowed. To also account for the accuracy of the perception of internal stimuli other than the heartbeat, Andor et al. [30] introduced a novel interoceptive accuracy task looking at the perception of spontaneous skin conductance fluctuations. In this task, phases with stable skin conductance (no nonspecific skin conductance fluctuations) thus representing the absence of internal arousal, as well as nonspecific skin conductance fluctuations (NSCF), representing current phasic sympathetic arousal, are recorded. Participants have to decide if an acoustic signal was preceded by either phasic arousal or a period of stable skin conductance. This method allows the use of signal detection methodology and thus the calculation of a perception bias to estimate whether participants spuriously perceive bodily symptoms (cf. Katzer et al. [31] for the concept of illusory bodily symptoms and its relevance to the understanding of somatic symptom disorder).

In an attempt to explore interoception as clinically relevant construct to tinnitus, we conducted an* a priori* power analysis based on effect sizes from a review paper on anxiety disorders [32]. Sample size was chosen to be able to detect a momentous, clinically relevant difference between the groups. We reasoned that, in order to establish interoception in tinnitus as a valid and meaningful construct, effect sizes should be comparable to, for example, panic disorder.

A multimethod assessment of interoception, including interoceptive sensibility through questionnaire measures and interoceptive accuracy through the Schandry task and the skin conductance task, was used. We hypothesized that a group with tinnitus sufferers exhibits higher levels of interoceptive sensibility and accuracy compared to a group of healthy controls. Based on findings in studies trying to better understand somatic symptom disorder such as health anxiety [28], we also expected an interoceptive bias and postulated that the tinnitus group shows a more liberal bias towards the perception of internal processes, for example, phasic sympathetic arousal, irrespective of its actual occurrence.

Tinnitus sufferers regularly associate stress with tinnitus [33]. For example, in a study by Baigi et al. [34], stress was related to worsening of the tinnitus. Hébert and Lupien [35] found higher cortisol levels in a tinnitus group compared to a control group after stress induction. Since stress appears to be associated with tinnitus, we hypothesized that the tinnitus group shows higher interoceptive accuracy under induced stress, whereas the performance of the control group should be less affected. To rule out a better performance based on an increased cardiac output due to the stress, we included a control condition where the participants had to reach elevated levels of cardiac output through movement on an ergometer.

## 2. Methods and Materials

### 2.1. Participants

Groups were matched with respect to age, gender, and level of education. Unexpectedly, the groups differ in the Body Mass Index (BMI) (Table 1). The study protocol was approved by the ethics committee of the Department of Psychology at the University of Münster and was conducted according to the Declaration of Helsinki. Recruitment was conducted through advertisements in local newspapers, an announcement on the institute's website, and the distribution of information brochures and posters throughout the university and in different locations in town. Participants were paid 20€ for their attendance. Exclusion criteria were high blood pressure, cardiac diseases, asthma, and pregnancy as the stress induction might have been disadvantageous for individuals showing any of these conditions. Pulsatile tinnitus, medication with cardiovascular or psychopharmacological effects, and any diagnosis of mental disorder were additional exclusion criteria. The absence of mental disorders was ensured by assessing all participants with the structured clinical interview for mental disorders for DSM-IV (SCID, German version; [36]).

### 2.2. Procedure

All potential participants were prescreened for the above-mentioned exclusion criteria via telephone. An e-mail including the study information sheet was sent to individuals meeting all inclusion criteria. On the day of the appointment, each participant gave written informed consent prior to participating in the experiments. The assessment started with the SCID to ensure absence of any mental disorder, which was the case for all participants.

#### 2.2.1. Questionnaires

Following the suggestion by Mehling et al. [37], different questionnaires to assess interoceptive sensibility were utilized. We used the Body Awareness Questionnaire (BAQ, [38]), a scale covering the perception of nonemotive, normal body processes, for example, rhythms of the body and anticipating body reactions. Furthermore, we used the first questionnaire dealing with interoception: the Private Body Consciousness Scale (PBCS, [39]), which measures a disposition to focus on internal processes, a sensitivity for bodily changes, and the awareness of interoceptive feedback. Additionally we handed out the Multidimensional Assessment of Interoceptive Awareness (MAIA, [40]), an eight-dimensional questionnaire covering noticing, notdistracting, not-worrying, attention regulation, emotional awareness, self-regulation, body listening, and trusting. For all three questionnaires, reliability and valditiy could be shown [38, 41, 42]. Positive affectivity and negative affectivity were measured with the Positive and Negative Affect Scale (PANAS, [43]) and somatization with the Screening for Somatoform Disorders (SOMS-7T, [44]). The tinnitus group additionally completed the Tinnitus Handicap Questionnaire (THQ, [45]) and the Tinnitus Handicap Inventory (THI, [46]) to quantify their tinnitus distress as well as visual analogue scales (VAS) covering the topics of perceived loudness, annoyance, distress, and handicap of their tinnitus.

#### 2.2.2. Skin Conductance Task

**Figure 1:**
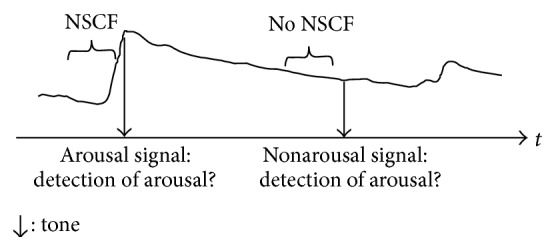
Description of the skin conductance task as measure of interoceptive accuracy as depicted in Andor et al. (2008). NSCF: nonspecific skin conductance fluctuation, *t* = time.

Skin conductance was measured with a Varioport (Becker Meditec, Karlsruhe, Germany) with a sampling rate of 16 Hz. Two silver/silver chloride electrodes with a contact surface area of 2 cm² to which isotonic paste was applied were used [47]. The electrodes were attached to the palm of the nondominant hand [48]. The Variotest system (Gerhard Mutz, Cologne, Germany) identified online periods of stable skin conductance (no NSCF) and periods of phasic sympathetic activation (NSCF), for a more detailed description: Andor et al. [30]. Participants were instructed to focus on their body arousal during the entire task and indicate, after each tone, whether a tone was preceded by an occurrence of body arousal (see Figure 1 for an illustration of the task procedure). The algorithm, whether the program was scanned for a stable phase or a fluctuation, was pseudorandomized with the restriction that the two different types of phases were not signaled more than two times in a row. The same sequence was used for all participants. The search window for a stable phase or a fluctuation was 150 s. If the intended event occurred within this time frame, the tone was presented; otherwise, no tone was presented and the program continued with the next trial. If more than five trials were missed, the subject was excluded from data analysis and the time window was shortened to 30 s. The latter intended to ensure that all participants started with the same feeling into the second task; for example, they did not notice that the task was cancelled. Usually participants are relaxed during this task and show only a few fluctuations in the skin conductance. In order to increase the arousal level of the participants, that is, provoke more fluctuations, two one-minute breaks were included in the task in which participants were asked to talk about their last vacation, book, or movie. As participants are usually more aroused in the beginning of an experimental session than in later phases, we chose to conduct the skin conductance task always first before the Schandry task.

#### 2.2.3. Schandry Task

In the second task, interoceptive accuracy was measured using the Schandry task [29]. Participants were instructed to count their heartbeat for an indicated amount of time. The electrocardiogram (ECG) was measured with a technical device (NeXus-10 Mark II, Mind Media BV, Herten, Netherlands) using three silver/silver chloride electrodes attached to the torso according to Einthoven lead II. The ECG was sampled at a rate of 256 Hz. The trials were presented with Presentation (Neurobehavioral Systems Inc., Berkeley, CA, USA).

**Figure 2:**
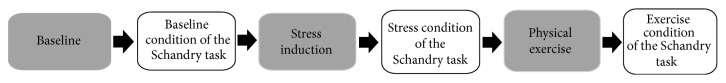
Procedure of the Schandry task.

Three within-subject conditions existed for this task: a baseline condition with the classic Schandry task, a condition following a social stress induction, and a control condition following physical exercise on an ergometer (see Figure 2). Each condition consisted of five consecutive trials of different length (20, 25, 30, 35, and 40 s) which were presented in randomized order. After each trial, participants had 10 s to report their heartbeat count to the investigator. A 30-second pause followed each trial. The beginning and the end of each trial were marked by a tone (onset tone: 800 Hz, 300 ms; offset tone: 500 Hz, 300 ms).

Before the baseline Schandry task, participants were given five minutes to get used to the ECG and afterwards filled out the good-and-bad mood and agitation-tranquility scale of the Multidimensional Questionnaire of Mental State (Mehrdimensionaler Befindlichkeitsfragebogen, MDBF, [49], cf. [50]) to report on their current mental state. Afterwards, the Schandry task was presented. Participants were asked to sit upright with their back of the hands resting on their thighs. This and the explicit instruction to avoid any other auxiliary means (e.g., measuring the pulse with the fingertips) were intended to ensure that the participants relied on their interoception solely. One test trial was conducted to make the participants familiar with the task. Then, the baseline Schandry task with its five trials was presented, followed by the stress induction. Here the participants performed the cognitive stress task of the Trier Social Stress Test [51]. For five minutes, participants had to repeatedly subtract 13, starting at 1022. They were told to do this mental arithmetic task as fast and as accurate as possible. In case of an error, participants were interrupted and told to start again at 1022. To further increase stress, the investigator said “Please calculate faster.” Moreover, participants were told that also the voice and the posture during this mathematical task would be analyzed and therefore he or she was videotaped during the task. To enhance stress levels through the additional factor of self-awareness, participants could see themselves on a screen. After the stress induction period, participants again filled out the MDBF. The second block of five trials of the Schandry task was presented, followed by another completion of the MDBF. Finally, the last condition of the Schandry task started: heartbeat perception after physical exercise (five minutes cycling on an ergometer). The investigator instructed the participant to either speed up or slow down their cycling to adjust their average heart rate to the heart rate measured in the stress induction phase. Finally, the third block of the Schandry task was conducted. At the end of the experiment participants were informed about the purpose of the experiment, including the function of the stress induction.

### 2.3. Analysis

#### 2.3.1. Skin Conductance Task

According to participants' ability to detect NSCFs, the sensitivity index *d*′ was calculated as follows: *Z*(hit rate) – *Z*(false alarm rate). If the hit rate equals the false alarms rate, the index is zero implicating low sensitivity. The higher *d*′ is, the better the participants were able to detect phasic internal arousal correctly. Furthermore, an index to quantify bias C as response behavior was calculated: −0.5 *∗* (*Z*(false alarm rate) + *Z*(hit rate)). It describes whether the participant had a conservative response behavior, that is, reporting more often no arousal than arousal, or a liberal one, that is, reporting more often arousal irrespective of its occurrence. The first is represented through an index higher than 0 and the latter below 0; an index around 0 reflects that there is no tendency, for example, a balanced answering behavior.

#### 2.3.2. Schandry Task

Data from the NeXus (including the triggers from Presentation) was imported to the Polyman program (Bob Kemp & Marco Roessen, Den Haag, Netherlands) to quantify the participants' heartbeat. Based on this data (recorded heartbeat) and the answers given by the participants (counted heartbeat) during the experiment, the heartbeat perception score was calculated (HBP, HBP = 1 − 1/5 ∑(|recorded heartbeats − counted heartbeats|/recorded heartbeats), (cf. [11, 52, 53])). The better the performance, that is, the accuracy of the given answers, the higher the HBP. The maximal value is 1.

## 3. Results

Twenty participants with chronic tinnitus (M = 42.8 years, SD = 13.1, 40% female) and twenty healthy control participants without tinnitus (M = 41.7 years, SD = 12.9, 40% female) were tested. Groups were matched with respect to age, gender, and level of education. Unexpectedly, the groups differ in the Body Mass Index (BMI); see Table 1.

### 3.1. Questionnaires

The *t*-test for independent samples revealed no significant differences between the two groups regarding the self-report measures of interoception, BAQ: *t*(38) = −0.70, *p* = 0.24; PBCS: *t*(38) = −0.08, *p* = 0.47; MAIA: *t*(38) = −0.61, *p* = 0.27. The same is true for the SOMS, *t*(38) = 0.12, *p* = 0.91, PANAS-PA, *t*(38) = −0.46, *p* = 0.65, and PANAS-NA, *t*(38) = 0.43, *p* = 0.67 (see Table 1).

### 3.2. Skin Conductance Task

**Figure 3:**
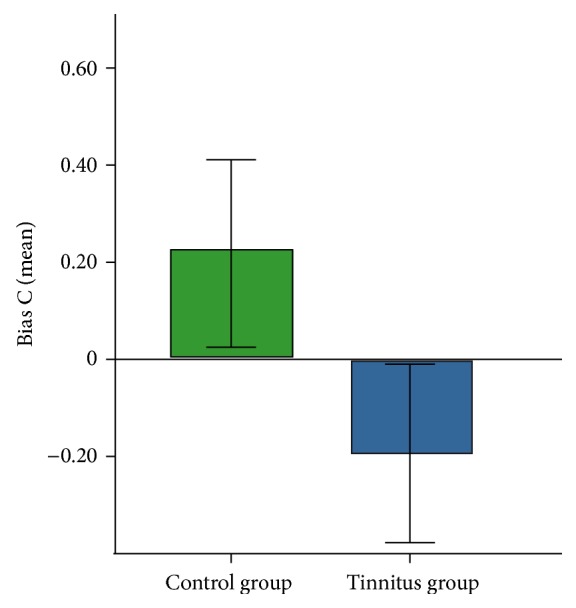
Mean score for bias C in the control and the tinnitus group. Error bars indicate the standard error.

Due to too few spontaneous skin conductance fluctuations (less than 5), 12 participants had to be excluded from the analysis, yielding 15 participants in the tinnitus group and 13 in the control group. We found no difference of the sensitivity index *d*′ between the groups in a *t*-test for independent samples, *t*(26) = 0.59, *p* = 0.28, and *d* = 0.22 (tinnitus group M = −0.14, SD = 1.30 and control group M = 0.16, SD = 1.51). A trend was found for the bias C: *t*(26) = 1.53, *p* = 0.07, *d* = 0.58 (see Figure 3, tinnitus group M = −0.19, SD = 0.71 and control group M = 0.22, SD = 0.70).

### 3.3. Schandry Task

**Figure 4:**
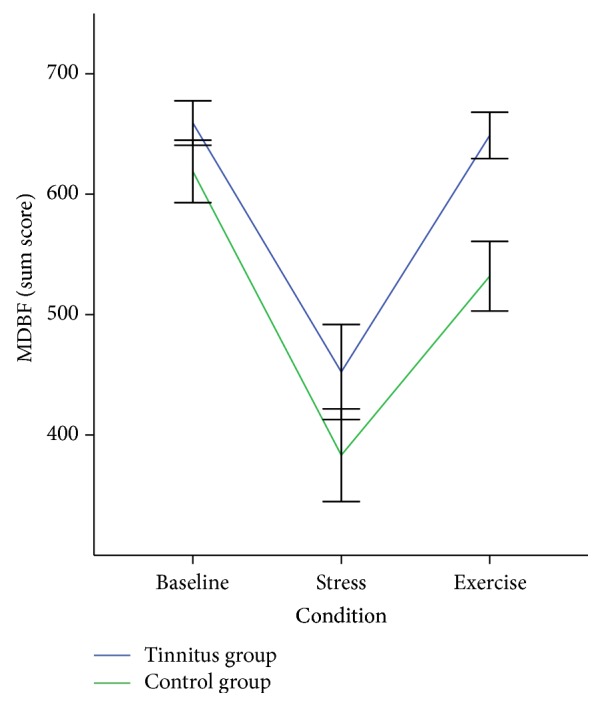
Current mood throughout the Schandry task conditions for both groups. Error bars indicate standard error. MDBF: Multidimensional Questionnaire of Mental State.

All participants were included in the analysis. In order to check if we successfully implemented the three conditions we compared the three consecutive current mental state scores. A repeated measures ANOVA with the factors* Condition* (baseline versus stress versus exercise) and* Group* (tinnitus versus control) showed a significant difference between the three conditions, *F*(2,76) = 53.12, *p* < 0.001, and *η* ^2^ = 0.67 (see Figure 4).

Simple contrasts revealed that the current mental state after the stress induction was significantly reduced compared to baseline, *F*(1,38) = 62.21, *p* < 0.001, and the exercise condition, *F*(1,38) = 74.26, *p* < 0.001. There was no significant interaction between group and condition, *F*(2,76) = 1.47, *p* = 0.24, and *η* ^2^ = 0.04.

Besides the self-report measure of mood, we also evaluated heart rate in the three different conditions. The lowest heart rate was found in the baseline condition (M = 73.04, SD = 14.01), followed by the stress (M = 85.90, SD = 17.53) and the exercise condition (M = 88.20, SD = 15.94). An ANOVA for repeated measure with the factors* Condition* and* Group* showed again a significant difference for the conditions for the heart rate values, *F*(2,76) = 60.68, *p* < 0.001, and *η* ^2^ = 0.62. Simple contrasts showed a significant difference in heart rate between baseline condition and both stress condition, *F*(1,38) = 45.59, *p* < 0.001, and exercise condition, *F*(1,38) = 106.11, *p* < 0.001, and a significant difference between the stress condition and the exercise condition, *F*(1,38) = 6.53, *p* = 0.02. There was no significant interaction of group *x* condition, *F*(2,76) = 0.05, *p* = 0.96, and *η* ^2^ = 0.00.

**Figure 5:**
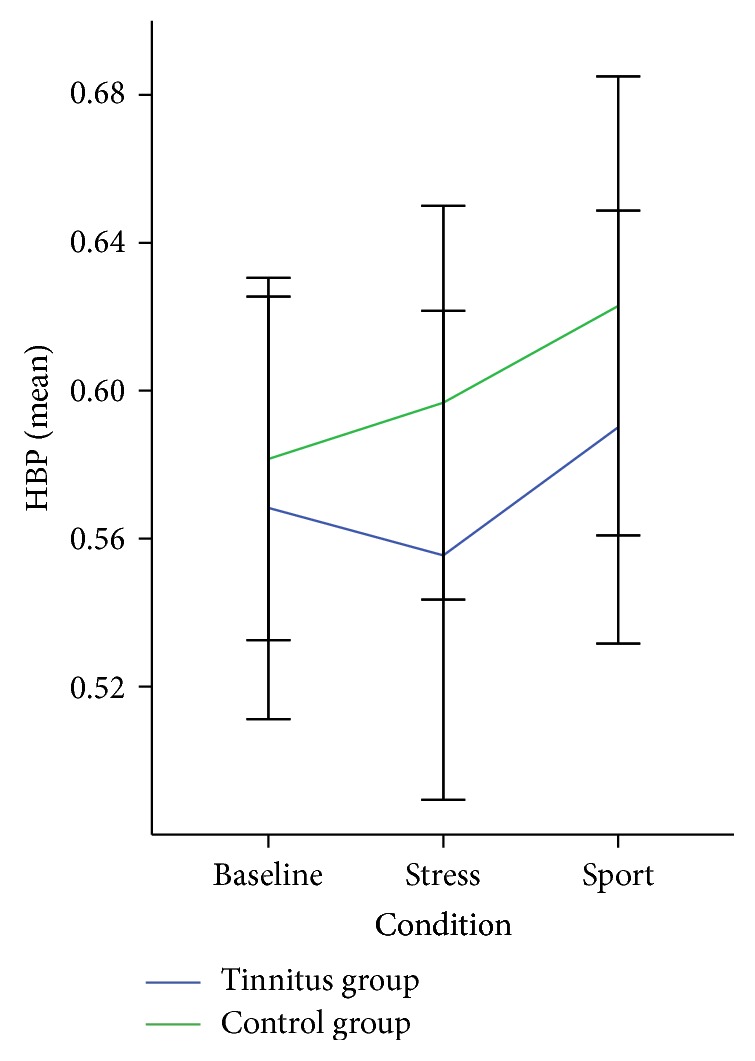
Heartbeat perception score (HBP) of the tinnitus and control group in the three conditions (baseline, stress, and exercise) of the Schandry task. Error bars indicate the standard error.

Using a repeated measures ANOVA on the HBP values with the factors* Condition* and* Group*, no main effect for groups with regard to HBP, *F*(2,76) = 1.90, *p* = 0.16, nor an interaction effect, *F*(2,76) = 0.30, *p* = 0.73, was found (see Figure 5).

### 3.4. Post Hoc Analysis

**Figure 6:**
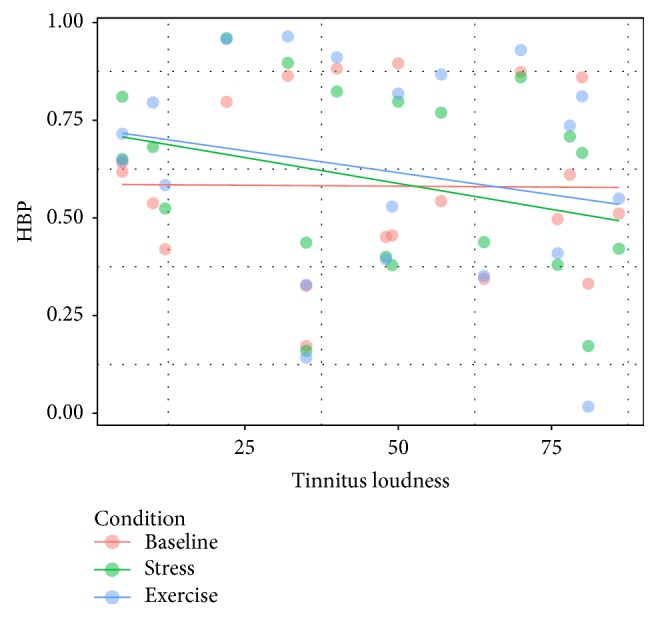
Correlation between the heart beat perception (HBP) score and tinnitus loudness. The correlation is plotted separately for the three conditions of the Schandry task. Colored lines represent the linear regression lines for each condition.

An analysis of covariance (ANCOVA) for the performance in the Schandry task in the three conditions within the tinnitus group was conducted, using tinnitus loudness as a covariate. Bonferroni correction for multiple testing was applied. This analysis revealed a significant difference between the three conditions when controlling tinnitus loudness, *F*(2,36) = 5.16, *p* = 0.02, as well as the interaction condition *x* loudness, *F*(2,36) = 4.39, *p* = 0.04 (see Figure 6). A simple linear regression analysis to predict the performance in the Schandry task for the stress condition compared to the baseline condition revealed a significant influence of tinnitus loudness, *F*(1,18) = 8.55, *p* < 0.01, and *R* ^2^ = 0.28. A marginally significant effect was found for the influence of loudness on the performance in the exercise condition, *F*(1,18) = 3.99, *p* = 0.06, and *R* ^2^ = 0.18. Quiet tinnitus went along with an enhanced performance in the Schandry task, especially in the stress and exercise condition, whereas loud tinnitus is accompanied with a decreased performance in the stress and exercise task.

A second ANCOVA for repeated measures for the performance in the Schandry task with BMI as covariate did not reach significance level, *F*(2,74) = 0.37, *p* > 0.99 (again Bonferroni corrected for multiple comparisons).

## 4. Discussion

We evaluated whether interoceptive sensibility and accuracy as key factors of interoception differed in a sample with chronic tinnitus and healthy control subjects. We found no clinically relevant differences between the groups, neither using questionnaires (interoceptive sensibility) nor using experimental tasks (the Schandry task and a skin conductance task, interoceptive accuracy). However, a trend in the bias measure C towards a more liberal perception of arousal, that is, a higher preparedness to expect internal arousal, in the tinnitus group was detected. Furthermore, tinnitus loudness influenced performance on the Schandry task in the chronic tinnitus group.

In order to detect a clinically relevant influence of interoception on tinnitus, we based our* a priori* power calculations on the averaged effect size from a review [32] for the Schandry task, which is the most field-tested and standardized task for interoception. However, our results show that changes in interoception in chronic tinnitus are not comparable to anxiety disorders.

Our hypothesis that the tinnitus group might perform better in interoceptive accuracy when stressed, that is, trying to roughly simulate the cooccurrence of stress and tinnitus onset, was not supported by the obtained experimental results. Given the comparable heart frequency, we assumed that the origin of the heart beat differences, stress or exercise, might have an influence on interoception. Yet there was no difference between performance in the two conditions, nor an interaction effect between group and condition. Heart beat elevation and the self-report of mental state after the stress induction reflect a successful manipulation. Yet we do not know how long the elevated stress level after the induction lasted. At least before the beginning of the exercise condition, the stress levels went back to normal. It might be worth to enhance the stress level more persistently or “refresh” the stress level between each trial in order to come to a final conclusion about the connection of stress level and interoceptive accuracy.

If we evaluate loudness of the tinnitus as a covariate for the Schandry task performance, we find a significant difference for the conditions in the Schandry task. The louder the tinnitus, the worse the heart beat perception performance in the stress and exercise condition. In the baseline condition, the cognitive load is lower and the cognitive resources are not yet depleted. Thus, it can be hypothesized that, with a quiet tinnitus perception, attention shifts are still possible as the participants were able to take away their attention from the tinnitus and focus on the task. If the tinnitus is especially loud, this might reduce the capacity to direct the attention away from the tinnitus towards perception of the heartbeat. This is in line with previous findings of difficulties of especially severe tinnitus sufferers on selective and divided attention [54, 55].

As the BMI negatively correlates with interoceptive accuracy for the heartbeat [56], the significant difference between the groups regarding this factor may have influenced the results as well. However, using BMI as covariate did not change our results.

The skin conductance task especially suffered from a low power: due to its novelty, effect sizes were difficult to estimate and in addition we encountered an unexpectedly significant number of dropouts. Furthermore, both groups had a low *d*′ score, representing guessing probability in this task. Whereas this finding is not completely surprising, given that in the two previous studies *d*′ scores in healthy control groups were also low, in our study the *d*′ scores were lower than what was previously found [28, 30]. Obviously, the task was too difficult for both groups and the especially low *d*′ scores render it unlikely that chronic tinnitus sufferers are especially adept at perceiving phasic sympathetic arousal as indexed by nonspecific skin conductance fluctuations.

Notwithstanding the bias C calculation is interesting. This finding adumbrates that the tinnitus group tends to perceive a bodily sensation, regardless of its actual physical occurrence. This perception bias might also apply to internal acoustic sensations and might be a starting point for a tinnitus sensation. Another possible explanation for the current results might be that people suffering from tinnitus may only have specifically increased interoception for internal acoustic processes which would not be detected through the measures used in the study at hand. Albeit we try to cover the concept of interoception as broad as possible, those measures might have been too coarse to detect this idea about specific and solely auditory interoception.

In contrast to these findings of interoceptive accuracy, another study found a reduced discrimination of external, electromagnetically evoked stimuli [57]. In the future, it might be interesting to investigate the relationship between extro- and interoception in tinnitus.

Overall our population was lowly distressed through their tinnitus. According to severity grading [58], 45% of our subjects had negligible tinnitus which is only audible in quiet surroundings, 40% a light tinnitus which can easily be ignored, and the rest mild tinnitus, where daily functioning is not impaired. The two more severe categories were not represented within our study. Through our screening for mental disorders, we might have likewise excluded highly distressed tinnitus sufferers as high distress in tinnitus is often accompanied by a mental disorder [59]. We would assume that in a high distressed group interoceptive processes might be more pronounced. This is also a key distinctive characteristic which varies between our study and the studies on interoception in mental disorders. In order to be diagnosed with a mental disorder, high distress and impairment are necessary; in the study at hand, we explicitly excluded participants based on this aspect.

Concluding, as first study in this field we tried to track down interoception in tinnitus. We took recent developments into consideration and systematically analyzed different aspects of interoception. In order to exclude confounders of interoception, we matched the two groups and profoundly screened for mental disorders. Despite our reasoning, we did not detect any main differences between a tinnitus group and a group of healthy controls regarding interoceptive accuracy and sensibility. If there are differences in the interoception between the two groups, the impact is not comparable to other disorders, for example, panic disorder and eating disorders. Yet we found that tinnitus sufferers might have a bias to perceive bodily symptoms irrespective of a physiological basis. Finally, we found that the loudness of tinnitus goes along with a decrease in performance in cognitive demanding tasks. We think it might be worth to further investigate the bias effect on the tinnitus population and to continue to complete the analysis of clinically relevant psychological variables influencing tinnitus and its distress.

## Figures and Tables

**Table 1:** Demographic description and mean scores of the questionnaires of tinnitus group and control group.

	Tinnitus group (*n* = 20)		Control group (*n* = 20)		*t*(38)	*p*
	M	SD	M	SD		
Age (in Years)	42.8	13.1	41.7	12.9	0.26	0.80
BMI (kg/m^2^)	25.0	3.9	22.3	3.2	2.32	0.03^*∗*^
Physical exercise per week (hours)	3.6	2.5	3.9	2.8	−0.34	0.74
Baseline heart rate (beats per minute)	75.8	15.7	70.3	11.9	1.26	0.22
BAQ	68.75	11.67	65.60	16.37	−0.70	0.24
PBCS	11.95	3.734	11.85	4.32	−0.08	0.47
MAIA	3.00	0.39	2.91	0.54	−0.61	0.27
SOMS	59.05	10.11	58.65	11.87	−0.46	0.65
PANAS-PA	30.65	5.68	31.50	6.09	0.43	0.67
PANAS-NA	11.85	1.39	11.55	2.84	0.12	0.91
THQ	22.18	16.01	—	—		
THI	23.80	13.73	—	—		

BMI: Body Mass Index, BAQ: Body Awareness Questionnaire, PBCS: Private Body Consciousness Scale, MAIA: Multidimensional Assessment of Interoceptive Awareness, SOMS: Screening for Somatoform Disorders, PANAS-PA: Positive Affect Scale of the Positive and Negative Affect Scale, PANAS-NA: Negative Affect Scale of the Positive and Negative Affect Scale, THQ: Tinnitus Handicap Questionnaire, THI: Tinnitus Handicap Inventory, ^*∗*^ *p* < 0.05.
